# Proton pump inhibitor use and risk of hepatic encephalopathy: A multicentre study

**DOI:** 10.1016/j.jhepr.2024.101104

**Published:** 2024-04-26

**Authors:** Simon Johannes Gairing, Chiara Mangini, Lisa Zarantonello, Elise Jonasson, Henrike Dobbermann, Philippe Sultanik, Peter Robert Galle, Joachim Labenz, Dominique Thabut, Jens Uwe Marquardt, Patricia P. Bloom, Mette Munk Lauridsen, Sara Montagnese, Christian Labenz

**Affiliations:** 1 Department of Internal Medicine I, University Medical Center of the Johannes Gutenberg-University, Mainz, Germany; 2 Cirrhosis Center Mainz (CCM), University Medical Center of the Johannes Gutenberg-University, Mainz, Germany; 3 Department of Medicine, University of Padova, Padova, Italy; 4 Department of Gastroenterology and Hepatology, Hospital of South West Jutland, Esbjerg, Denmark; 5 Department of Medicine I, University Hospital Schleswig-Holstein, Lübeck, Germany; 6 Service d'hépato-gastroentérologie, Sorbonne Université, Hôpital Pitié-Salpêtrière Assistance Publique Hôpitaux de Paris, Paris, France; 7 Department of Medicine, Diakonie Hospital Jung-Stilling, Siegen, Germany; 8 Department of Internal Medicine, Division of Gastroenterology and Hepatology, University of Michigan, Ann Arbor, MI, United States; 9 Chronobiology Section, Faculty of Health and Medical Sciences, University of Surrey, Guildford, UK

**Keywords:** Covert hepatic encephalopathy, Decompensated cirrhosis, Psychometric hepatic encephalopathy score, Acid suppression

## Abstract

**Background & Aims:**

Data on the association between proton pump inhibitor (PPI) use and hepatic encephalopathy (HE) are conflicting, and data from multicentre studies are scarce. The aim of this study was to dissect the potential association between PPI use and minimal (MHE) and overt HE (OHE).

**Methods:**

Data from patients with cirrhosis recruited at seven centres across Europe and the US were analysed. MHE was defined by the psychometric hepatic encephalopathy score (PHES). PPI use was recorded on the day of testing with PHES. Patients were followed for OHE development and death/liver transplantation.

**Results:**

A total of 1,160 patients with a median MELD of 11 were included (Child-Pugh stages: A 49%/B 39%/C 11%). PPI use was noted in 58% of patients. Median follow-up time was 18.1 months, during which 230 (20%) developed an OHE episode, and 224 (19%) reached the composite endpoint of death/liver transplantation. In multivariable analyses, PPI use was neither associated with the presence of MHE at baseline nor OHE development during follow-up. These findings were consistent in subgroup analyses of patients with Child-Pugh A or B cirrhosis and after excluding patients with a history of OHE. PPI use was also not associated with a higher risk of OHE, neither in patients with an indication for treatment nor in patients without an indication.

**Conclusions:**

PPI use is not associated with a higher risk of HE in patients with cirrhosis. Based on these findings, at present, a prescription should not be prohibited in case of a generally accepted indication.

**Impact and implications:**

Data on the association between proton pump inhibitor (PPI) use and hepatic encephalopathy (HE) are conflicting. In this study, PPI use was not associated with a higher risk of minimal HE at baseline or overt HE during follow-up in patients with cirrhosis. Based on these findings, prescription of a PPI for a generally accepted indication should not be prohibited in patients with cirrhosis.

## Highlights

• 58% of patients with cirrhosis had a PPI prescription, of whom only 26.5% had a generally accepted indication.
• PPI use was not associated with an increased risk of minimal hepatic encephalopathy at study inclusion.
• PPI use was not associated with an increased risk of overt hepatic encephalopathy during follow-up.

## Graphical abstract

**Figure:**
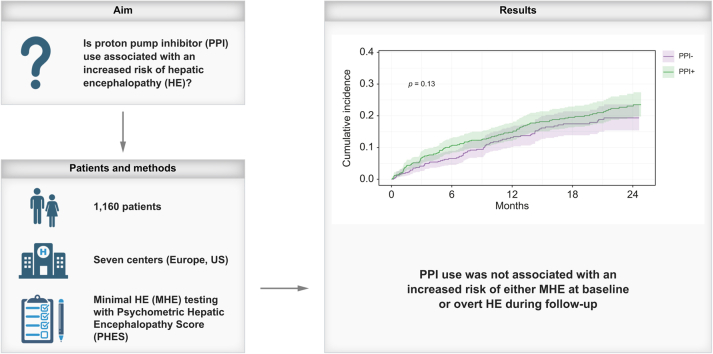

## Introduction

Cirrhosis is prevalent worldwide and is the main cause of about 1.32 million deaths per year.^1^ One of the most severe complications of cirrhosis is hepatic encephalopathy (HE) – a neurophysiological complication that negatively impacts patients’ quality of life and poses a huge burden for caregivers.^2^^,^^3^ According to the current guidelines, HE can be divided into covert HE and overt HE (OHE).^4^ Covert HE is an umbrella term combining the two lowest HE grades — minimal HE (MHE) and HE grade 1 according to the West Haven Criteria, while OHE covers grades 2–4. The prevalence of MHE correlates with the stage of liver disease and ranges from <25% in patients with Child-Pugh (CP) A cirrhosis to >50% in patients with CP C.^5^ The incidence rates of OHE are less well studied and have been reported to be approximately 10% and 25% per year in patients with CP A and B cirrhosis, respectively.^6^ Given that MHE is associated with a poorer quality of life and a higher risk of OHE and that OHE is associated with a poorer prognosis and a huge burden for caregivers,^2^^,^^3^^,^^7^^,^^8^ it is of the utmost importance to identify potentially modifiable factors associated with a higher risk of HE.

In recent years, several studies have investigated the association between PPI use and HE, and most found a significant association between PPI intake and the risk of HE. PPI might modify the intestinal microbiome, which may increase the production of ammonia and other toxic compounds involved in the pathogenesis of HE.^9^^,^^10^ Thus, the hypothesis arose that there might be an association between PPI use and HE. However, most studies on this topic had limitations in terms of their design (cross-sectional and relying on ICD-codes,^11^ a lack of MHE testing at baseline,^12^ or only including a single-centre cohort^13^). Currently, only two studies investigating the association between PPI use and HE considered MHE testing at baseline.^13^^,^^14^ Surprisingly, the results were conflicting: the study by Nardelli *et al.* found a robust association between PPI use and MHE as well as OHE, while the study by Acharya *et al.* found no association.^13^^,^^14^ Both studies were based on cohorts from one (Nardelli *et al.*) or two (Acharya *et al.*) centres and were not adequately powered for subgroup analyses. Therefore, it was the aim of this study to investigate a potential association between PPI use and M/OHE in a large multinational cohort of patients with cirrhosis and to conduct subgroup analyses according to the severity of cirrhosis.

## Patients and methods

This retrospective study analysed data on 1,160 patients who underwent MHE testing with PHES at the following centres with expertise in diagnosing MHE: Mainz, Siegen, Lübeck (all Germany), Paris (France), Padua (Italy), Esbjerg (Denmark), and Ann Arbor (Michigan, US). The details of this study are also described elsewhere.^5^^,^^15^ At each centre, the primary aetiology of the underlying liver disease was determined according to clinical, serological, and histological findings. Diagnosis of cirrhosis was established by histology, conclusive appearance on ultrasound, elastography or radiological imaging, endoscopic features of portal hypertension, or medical history. The severity of liver disease was determined by calculating the model for end-stage liver disease (MELD), CP, and the albumin-bilirubin (ALBI) scores.[16], [17], [18] MELD scores were calculated centrally using the original MELD formula of Kamath *et al.* The three ALBI grades were defined by cut-offs of ≤-2.60 (grade 1), >-2.60 to ≤-1.39 (grade 2), and >-1.39 (grade 3).

Patients were not included in this study if they had a history of any other disease leading to mental alterations (*e.g*., dementia or a history of stroke). At some centres, patients with self-reported ongoing (mild) alcohol consumption were included, provided that they were not under the effects of alcohol during testing with PHES. Other exclusion criteria varied between centres and are detailed for each centre elsewhere.^5^

### PPI use

In all patients, PPI use at study inclusion was recorded. Additionally, we recorded type of PPI (pantoprazole, omeprazole, esomeprazole, rabeprazole, lansoprazole) and the underlying indication for treatment, if there was one. Established indications were as follows: gastroesophageal reflux disease or symptoms of reflux, gastric or duodenal ulcer disease, and indication for ulcer prophylaxis.

### Diagnosis of MHE

All patients were examined at the respective hospitals by an experienced hepatologist to rule out clinical signs of OHE. For each patient, PHES was performed to diagnose MHE. PHES is a paper-and-pencil testing battery including five subtests (number connection test A and B, serial dotting test, digit symbol test and line tracing test). PHES was scored at each centre using the validated country-specific norms (Germany,^19^ US,^20^ Italy,^21^ France^22^). The Danish centre used the German norms. A score <- 4 was considered diagnostic of MHE for centres from Germany, France and Denmark, while the centres from Italy and the US used a score ≤-4.

### Follow-up evaluation

Patients were followed during regular clinic visits or via electronic chart review at the respective centres for the occurrence of OHE. The presence of OHE was diagnosed after a detailed neurological examination according to the West Haven criteria by an experienced hepatologist. Moreover, patients were followed for the occurrence of death or liver transplantation. Patients who did not reach the endpoint of death/liver transplantation were censored at the date of last contact.

### Ethics

The study was conducted in accordance with the ethical guidelines of the 1975 Declaration of Helsinki (7^th^ revision, 2013). For this study, we used anonymised electronic medical records without directly identifiable data. According to German regulations and the recommendations of the Ethics Committee of the Landesärztekammer Rheinland-Pfalz, no ethical approval is required for this type of study. Anonymised data were analysed as aggregates with no protected health information available. For the subset of patients with data recorded in a prospective setting, the respective study protocols were approved by the Ethics Committees of the respective centres, and all patients provided informed consent for participation.

### Statistical analysis

Statistical analysis was performed with R 4.1.3 (R Core Team (2022). R: A language and environment for statistical computing. R Foundation for Statistical Computing, Vienna, Austria. URL https://www.R-project.org/), RStudio version 2023.9.1.494 (Posit team (2023). RStudio: Integrated Development Environment for R. Posit Software, PBC, Boston, MA. URL http://www.posit.co/.) and IBM SPSS Statistic Version 29 (Armonk, NY: IBM Corp.). Baseline characteristics were calculated with the {table 1} R package (v1.4.3; Benjamin Rich 2023). Continuous data are given as median with range, and pairwise comparisons were performed with the Mann-Whitney *U* test. Categorical variables are shown as frequencies and percentages, and a chi-squared test was calculated for comparison of two or more groups. Median follow-up time was calculated using the reverse Kaplan-Meier method ({prodlim} R package (v2019.11.13, Thomas A. Gerds 2019)).

**Table 1:** Demographics and clinical characteristics of the study cohort.

	Total cohort (N = 1,160)	PPI- (n = 491)	PPI+ (n = 669)
Age (years)			
Median [Min, Max]	60.0 [23.0, 87.0]	59.0 [23.0, 87.0]	60.0 [26.0, 86.0]
Missing	1 (0.1%)	1 (0.2%)	0 (0%)
Gender			
Male	758 (65.3%)	326 (66.4%)	432 (64.6%)
Female	402 (34.7%)	165 (33.6%)	237 (35.4%)
Etiology			
Alcohol	553 (48.0%)	236 (48.3%)	317 (47.8%)
Viral	174 (15.1%)	83 (17.0%)	91 (13.7%)
Others/mixed	425 (36.9%)	170 (34.8%)	255 (38.5%)
Missing	8 (0.7%)	2 (0.4%)	6 (0.9%)
MELD score			
Median [Min, Max]	11.0 [6.00, 34.0]	10.3 [6.00, 33.0]	11.2 [6.00, 34.0]
Missing	66 (5.7%)	27 (5.5%)	39 (5.8%)
Child-Pugh			
A	539 (49.4%)	262 (56.7%)	277 (44.1%)
B	429 (39.4%)	156 (33.8%)	273 (43.5%)
C	122 (11.2%)	44 (9.5%)	78 (12.4%)
Missing	70 (6.0%)	29 (5.9%)	41 (6.1%)
ALBI grade			
1	210 (24.8%)	113 (32.2%)	97 (19.6%)
2	478 (56.4%)	179 (51.0%)	299 (60.3%)
3	159 (18.8%)	59 (16.8%)	100 (20.2%)
Missing	313 (27.0%)	140 (28.5%)	173 (25.9%)
MHE (PHES)			
MHE-	775 (66.8%)	348 (70.9%)	427 (63.8%)
MHE+	385 (33.2%)	143 (29.1%)	242 (36.2%)
History of ascites			
No	506 (44.3%)	235 (48.6%)	271 (41.2%)
Yes	636 (55.7%)	249 (51.4%)	387 (58.8%)
Missing	18 (1.6%)	7 (1.4%)	11 (1.6%)
History of OHE			
No	803 (70.6%)	355 (74.0%)	448 (68.1%)
Yes	335 (29.4%)	125 (26.0%)	210 (31.9%)
Missing	22 (1.9%)	11 (2.2%)	11 (1.6%)
Sodium (mmol/L)			
Median [Min, Max]	138 [116, 146]	139 [118, 145]	138 [116, 146]
Missing	202 (17.4%)	89 (18.1%)	113 (16.9%)
Creatinine (mg/dl)			
Median [Min, Max]	0.848 [0.320, 7.51]	0.826 [0.385, 4.15]	0.870 [0.320, 7.51]
Missing	76 (6.6%)	34 (6.9%)	42 (6.3%)
Bilirubin (mg/dl)			
Median [Min, Max]	1.24 [0.100, 33.3]	1.20 [0.175, 29.1]	1.30 [0.100, 33.3]
Missing	78 (6.7%)	33 (6.7%)	45 (6.7%)
Albumin (g/L)			
Median [Min, Max]	35.0 [14.0, 52.0]	36.0 [14.0, 52.0]	34.9 [15.0, 51.2]
Missing	223 (19.2%)	97 (19.8%)	126 (18.8%)
INR			
Median [Min, Max]	1.22 [0.800, 3.30]	1.20 [0.900, 3.30]	1.26 [0.800, 3.23]
Missing	113 (9.7%)	51 (10.4%)	62 (9.3%)
Lactulose			
No	723 (62.4%)	342 (69.7%)	381 (57.0%)
Yes	436 (37.6%)	149 (30.3%)	287 (43.0%)
Missing	1 (0.1%)	0 (0%)	1 (0.1%)
Rifaximin			
No	864 (74.5%)	384 (78.2%)	480 (71.9%)
Yes	295 (25.5%)	107 (21.8%)	188 (28.1%)
Missing	1 (0.1%)	0 (0%)	1 (0.1%)
Reason for PPI treatment∗			
Reflux/GERD			71 (10.6%)
Ulcer disease			38 (5.7%)
Dyspepsia			30 (4.5%)
Ulcer prophylaxis			24 (3.6%)
Mixed/others			14 (2.1%)
PPI indication			
No			492 (73.5%)
Yes			177 (26.5%)
PPI type			
Pantoprazole			382 (59.7%)
Esomeprazole			29 (4.5%)
Lansoprazole			115 (18.0%)
Omeprazole			100 (15.6%)
Rabeprazole			13 (2.0%)
No information on type			1 (0.2%)

∗ 73.5% had no PPI indication. ALBI, albumin-bilirubin; GERD, gastroesophageal reflux disease; MELD, model for end-stage liver disease; MHE, minimal hepatic encephalopathy; OHE, overt hepatic encephalopathy; PPI, proton pump inhibitor.

To identify variables associated with the prevalence of MHE, we conducted multivariable logistic regression analyses, including established risk factors for MHE and PPI use.

The {tidycmprsk} R package (v0.2.0, Daniel D. Sjoberg and Teng Fei 2022) was used for cumulative incidence functions for competing risk analyses and Fine and Gray competing risk regression analysis. For this purpose, a multi-state model with three states was used (0: alive without liver transplantation and no OHE event at the end of follow-up, 1: alive with an OHE event during follow-up and without liver transplantation prior to the event, 2: death or liver transplantation during follow-up without OHE event prior to death or liver transplantation). Differences between groups in cumulative incidence functions were calculated with Gray's test. Follow-up time was defined from study inclusion to censoring, OHE, death, or transplantation. Multivariable models were adjusted for biologically plausible and established risk factors for OHE. In addition, univariable competing risk regression was performed, and variables with a univariable *p* <0.05 were included in a multivariable model (plus PPI). Missing data were excluded from the analyses (complete case analysis).

## Results

In total, data from 1,160 patients with cirrhosis were available for analysis (Fig. S1). The cohort was predominantly male (65.3%) with a median age of 60 years (range 23-87). A history of OHE was noted in 335 patients (29.4%), and MHE, according to PHES, was present in 385 patients (33.2%). Additional demographics and clinical characteristics are displayed in Table 1. In addition, demographics and clinical characteristics of patients without a history of overt HE, as well as characteristics of the patients from the respective centres, are shown in Tables S1 and S2, respectively.

### Comparison of PPI users and non-users

PPI use was noted in 58% of the patients at study inclusion. The most frequently prescribed PPI was pantoprazole (59.7%) followed by lansoprazole (18.0%) and omeprazole (15.6%). Only 26.5% of the patients with a PPI prescription had a hard indication for treatment. Details on the distribution of PPI and the indications are displayed in Table 1, Tables S1, and S2. Compared to PPI non-users, PPI users had a higher MELD score and a higher rate of cirrhosis-related complications.

### PPI use and the presence of MHE

Comparisons of the characteristics of patients with and without MHE are provided in Table S3. Patients with MHE were in a more severe stage of cirrhosis as described by a higher MELD score or CP stage. Notably, PPI use was more frequent in patients with than without MHE (63% *vs.* 55%, *p* = 0.024). To assess the independent association between PPI use and the presence of MHE, a multivariable logistic regression model based on established and biologically plausible risk factors for MHE (albumin, MELD, history of OHE, gender, age) and PPI use was constructed. There was no significant association between PPI use and MHE in the total cohort nor in the sub-cohort of patients without a history of OHE (Table 2).

**Table 2:** Multivariable logistic regression analysis for the association between variables and MHE prevalence in the total cohort and in patients without a history of OHE.

Variables	Total cohort			Patients without a history of OHE		
	OR	95% CI	*p* value	OR	95% CI	*p* value
PPI use	1.07	0.80–1.43	0.7	1.02	0.72–1.46	0.99
Albumin (g/L)	0.94	0.92–0.97	**<0.001**	0.95	0.93–0.98	**<0.001**
MELD	1.02	0.99–1.05	0.3	1.05	1.01–1.09	**0.018**
History of OHE	1.80	1.29–2.38	**<0.001**	—	—	—
Gender	0.70	0.51–0.94	**0.018**	0.60	0.42–0.86	**0.006**
Age (years)	1.02	1.01–1.03	**0.029**	1.02	0.99–1.03	0.09

Patients with missing data were excluded from analysis (complete case analysis). *P* values in bold show significant values. MELD, model for end-stage liver disease; MHE, minimal hepatic encephalopathy; OHE, overt hepatic encephalopathy; OR, odds ratio; PPI, proton pump inhibitor.

### PPI use and the development of OHE

All patients were followed for the development of the first OHE episode after enrolment or death/liver transplantation. Median follow-up time was 18.12 months (95% CI 17.82–18.44). In total, 230 (19.8%) patients experienced at least one OHE episode. Additionally, 224 (19.3%) patients died or received a liver transplantation before an episode of OHE.

**Fig. 1:**
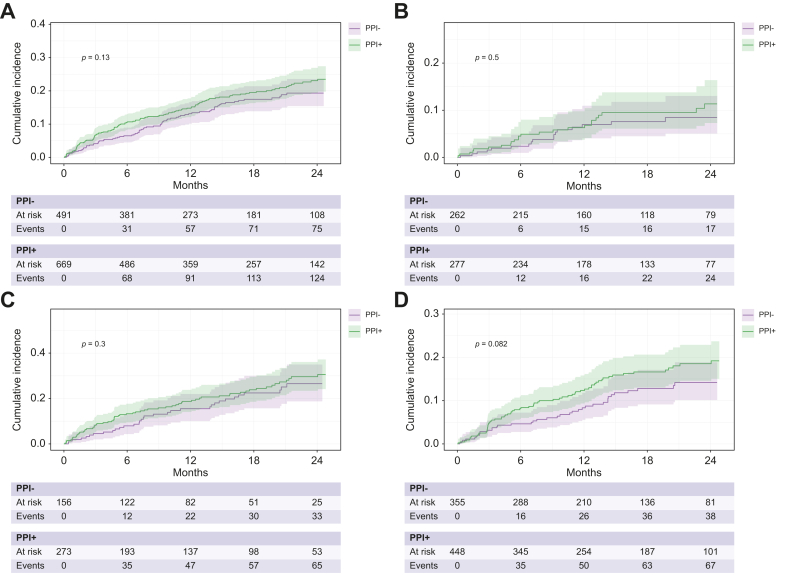
Cumulative incidence of overt HE (OHE). A three-state model was built with death and LTX as competing events. Differences between groups were calculated with Gray’s test. Cumulative OHE incidence in patients with vs without PPI use in **(A)** all patients (p = 0.13), **(B)** patients with Child-Pugh A (p = 0.5), **(C)** patients with Child-Pugh B (p = 0.3), and **(D)** patients without a history of OHE (p = 0.082). Abbr.: PPI, proton pump inhibitor.

In the total cohort, the cumulative OHE incidences did not differ significantly between PPI users and non-users (*p* = 0.13, Fig. 1A). To identify variables independently associated with the development of OHE, we fitted multivariable regression models using the method of Fine and Gray under consideration of the competing events death or liver transplantation. Again, established and biologically plausible risk factors for OHE, as well as PPI use, were included in the models. In the total cohort, PPI use was not associated with a higher risk of OHE in multivariable regression analysis (subdistribution hazard ratio [sHR] 1.13, 95% CI 0.81–1.59, *p* = 0.5; Table 3). This finding remained robust in a separate model including lactulose or rifaximin use at study inclusion (PPI use: sHR 1.08, 95% CI 0.77–1.51, *p* = 0.7) (Table S4). In addition, we fitted a multivariable competing risk regression model including only variables with a *p* value <0.05 in univariable analysis (Table S5 and Table S6). PPI use was not significantly associated with the development of OHE in univariable analysis (sHR 1.23, 95% CI 0.94–1.60, *p* = 0.13), but was nonetheless included in the multivariable model. Here, PPI use showed no significant association (sHR 1.16, 95% CI 0.82–1.64, *p* = 0.4).

**Table 3:** Multivariable Fine and Gray regression analysis for OHE development.

Variable	Total cohort				Child-Pugh A				Child-Pugh B			
	N	sHR	95% CI	*p* value	N	sHR	95% CI	*p* value	N	sHR	95% CI	*p* value
PPI use												
No	381	—	—		214	—	—		133	—	—	
Yes	527	1.13	0.81–1.59	0.5	220	1.63	0.84–3.16	0.2	237	1.12	0.71–1.76	0.6
MHE												
No	599	—	—		331	—	—		211	—	—	
Yes	309	1.33	0.94–1.87	0.11	103	1.02	0.44–2.33	>0.9	159	1.90	1.21–2.99	**0.005**
MELD	908	1.06	1.03–1.10	**<0.001**	434	1.18	1.03–1.34	**0.016**	370	1.05	0.99–1.11	0.10
Albumin (g/L)	908	0.93	0.91–0.96	**<0.001**	434	0.89	0.81–0.97	**0.011**	370	0.96	0.93–1.00	0.062
History of OHE												
No	661	—	—		362	—	—		229	—	—	
Yes	247	2.00	1.43–2.79	**<0.001**	72	4.26	2.16–8.37	**<0.001**	141	1.70	1.09–2.66	**0.020**
Age (years)	908	1.02	1.00–1.03	0.067	434	1.00	0.97–1.03	>0.9	370	1.02	1.00–1.04	**0.045**

Patients with missing data were excluded from analysis (complete case analysis). *P* values in bold show significant values. MELD, model for end-stage liver disease; MHE, minimal hepatic encephalopathy; OHE, overt hepatic encephalopathy; PPI, proton pump inhibitor; sHR, subdistribution hazard ratio.

Given that OHE incidence differs markedly between patients with CP A and B cirrhosis, we conducted subgroup analyses in these two groups. We did not conduct a subgroup analysis in patients with CP C due to the low case and event numbers. PPI use was not associated with a higher risk of OHE in patients with CP A or B cirrhosis, neither in univariable nor in multivariable analyses (Fig. 1B,C, Table 3 and Table S4).

Given that patients with a history of OHE are at higher risk for recurrent OHE, we repeated our analyses in the subgroup of patients without a history of OHE (n = 803) and in the subgroup of patients without a history of OHE and no prescription of either lactulose or rifaximin (n = 468). Again, there was no difference between PPI users and non-users in the cumulative OHE incidences or in multivariable regression analysis (Fig. 1D and Fig. S2, Table 4, Tables S7, and S8).

**Table 4:** Multivariable Fine and Gray regression analysis for OHE development in patients without a history of OHE.

Variable	N	sHR	95% CI	*p* value
PPI use				
no	288	—	—	
yes	373	1.33	0.80–2.19	0.3
MHE				
no	467	—	—	
yes	194	1.93	1.21–3.07	**0.006**
MELD	661	1.07	1.03–1.12	**0.002**
Albumin (g/L)	661	0.92	0.88–0.95	**<0.001**
Age (years)	661	1.02	1.00–1.04	0.067

Patients with missing data were excluded from analysis (complete case analysis). P-values in bold show significant values. MELD, model for end-stage liver disease; MHE, minimal hepatic encephalopathy; OHE, overt hepatic encephalopathy; PPI, proton pump inhibitor; sHR, subdistribution hazard ratio.

**Fig. 2:**
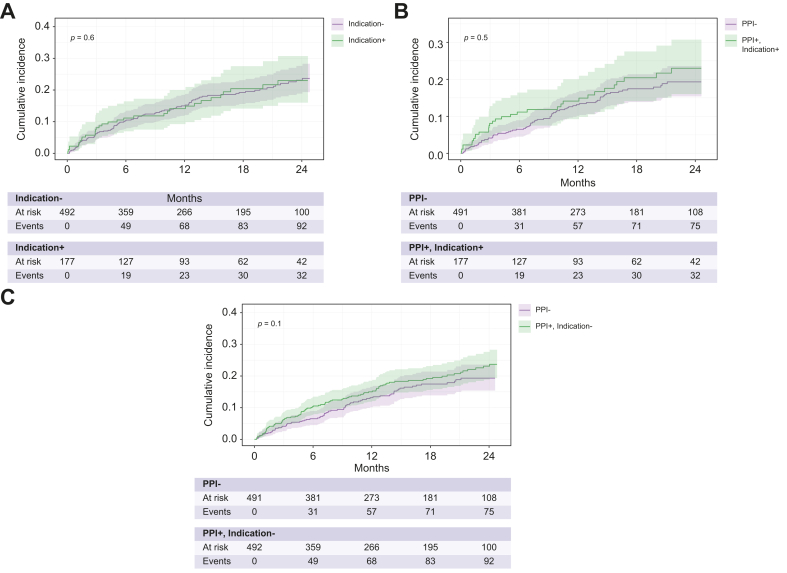
Cumulative incidence of overt HE (OHE). Cumulative OHE incidence in (A) patients with vs without an indication for PPI use (p = 0.6), (B) patients with an indication for PPI use and those without PPI use (p = 0.5), (C) patients with PPI use but no indication and those without PPI use (p = 0.1). Abbr.: PPI, proton pump inhibitor.

We also analysed cumulative OHE incidences in patients with and without an indication for PPI treatment. There was no significant difference between the cumulative OHE incidences either between patients with or without an indication for PPI use, nor between patients without an indication for PPI use and those without PPI use or those with an indication for PPI use and those without PPI use (Fig. 2).

## Discussion

In this study, we provide evidence against an association between PPI use and the presence of MHE or the development of OHE in patients with cirrhosis. This finding held true regardless of CP stage, and PPI indication. Factors associated with a higher OHE risk were related to worse liver function – MELD, albumin, and a history of OHE – as previously shown in this cohort.^15^ Our findings fuel the debate on whether or not PPI use is associated with complications of cirrhosis.^23^^,^^24^

PPIs are among the most frequently prescribed medications in cirrhosis. In our cohort, nearly 60% used PPI, of whom three out of four had no valid and documented indication. A similar observation was made in another study.^25^ Overall, the data illustrates an overuse, which is inexpedient irrespective of its effects on the prognosis of patients with cirrhosis. Two prior studies found an association between PPI use and adverse events in patients with cirrhosis,^26^^,^^27^ but high-quality data on the association between PPI use and HE are scarce. Important studies arguing for an association between PPI use and HE were published by Dam *et al.* and Nardelli *et al.*^12^^,^^13^ Acharya *et al.* analysed data from a large cohort of patients with MHE testing at baseline using PHES. In contrast to Dam *et al.* and Nardelli *et al.* and similarly to our findings, they found no association between PPI use and MHE or OHE during follow-up.^14^ However, the follow-up analysis in Acharya’s study only included patients with prior OHE, and the cohort mainly consisted of males. Our study expands the existing literature by providing data from a large multicentre cohort in which 70% of patients were OHE-naïve, all with available PHES testing at baseline and with sufficient power to detect associations between PPI use and HE. Our data argue against an association between PPI use and HE and conflict with most published studies on this topic.

This discrepancy might be partly explained by publication bias affecting the publication of negative studies. Additionally, some studies might be biased by confounding by indication for a PPI prescription, as recently described in a secondary analysis of the ATTIRE trial.^28^ Here, China *et al.* found that patients with PPI use had a significantly different clinical course (*e.g*. significantly higher number of suspected variceal bleeds in the PPI group) compared to those not prescribed PPIs. This fact may explain the significant association between PPI use and HE grade III/IV during hospitalisation in their study. Supporting this assumption, the authors found no association between PPI use and potential pathophysiological links, such as the degree of bacterial translocation or systemic inflammation.^28^

An important strength of our study, separating it from the one with a similar design published by Nardelli *et al.*,^13^ is our large sample size, allowing for good power across sub-analyses that all consistently showed no association between PPI use and HE. Our conclusions are further strengthened by a low number of randomly missing data points and the inclusion of only patients with full datasets in multivariable analyses.

The limitations of our study were that although most patients were prospectively recruited in the respective centres, the collection of PPI data and the subsequent analyses were retrospective with related weaknesses. The most important limitation is the lack of information on the start and duration of PPI use. Our data include information on PPI use at the time of psychometric testing and the indications were reviewed in the patient charts. It could be possible that PPI use is associated with the occurrence of adverse events in a time-dependent manner in patients with cirrhosis, which cannot be sufficiently analysed in this current study. Additionally, it could be possible that some patients might be resistant to the potentially harmful effects of PPI, if such effects exist. This could only be sufficiently addressed by implementing a “new user design” only including patients with a PPI prescription at the day of cognitive testing and subsequent testing during follow-up. However, these data are not available in the current study. Given the aforementioned limitations, we are unable to apply causal inference methods in this observational dataset. Therefore, it has to be acknowledged that our study can only detect associations and is unable to prove causality. Last, although the patients have a prescription for PPI, patients may not be taking them. Here, epidemiological studies, including prescription time data from prescription databases and actual drug purchase databases, might be more accurate.

Nevertheless, our data support that PPIs in a primarily OHE-naïve cohort might not be associated with an increased risk of OHE. However, the issue of causality can only be clarified by a randomized and prospective study, which is currently recruiting as part of the STOPPIT trial.^29^

In conclusion, we provide evidence against an association between PPI use and the presence of MHE and OHE development. This observation applied regardless of liver disease severity and PPI indication. Although PPI should only be prescribed when indicated, these data suggest that PPI use should not be restricted in patients with cirrhosis with a solid indication.

## Abbreviations

ALBI, albumin-bilirubin; CP, Child-Pugh; HE, hepatic encephalopathy; HR, hazard ratio; MELD, model for end-stage liver disease; MHE, minimal hepatic encephalopathy; OHE, overt hepatic encephalopathy; PHES, psychometric hepatic encephalopathy score; PPI, proton pump inhibitor; sHR, subdistribution hazard ratio.

## Financial support

This work was not supported by any grant or funding source.

## Conflicts of interest

Simon Johannes Gairing: Travel expenses: Ipsen and Gilead. Joachim Labenz: Consulting: Alfaasigma, Norgine. Lecture fees: Norgine. Peter R. Galle: Lecture fees and consulting: Merz Pharmaceuticals. Patricia P. Bloom: Research grant from Vedanta Biosciences. Consultant for Nexilico. Dominique Thabut: Lecture fees: Alfasigma, Abbvie, Gilead. Christian Labenz: Travel expenses and consulting: Norgine, Merz Pharmaceuticals. Lecture fees: Norgine, Merz Pharmaceuticals. Research grants: Norgine, Merz Pharmaceuticals. All other authors disclose no potential financial or non-financial conflict of interests regarding this study.

Please refer to the accompanying ICMJE disclosure forms for further details.

## Authors’ contributions

Performed research: S.J.G., C.M., L.Z., E.J.N., H.D., P.B., P.S., P.R.G., J.L., D.T., J.U.M., M.M.L., S.M., C.L. Contributed to acquisition of data: S.J.G., C.M., L.Z., E.J.N., H.D., P.B., P.S., P.R.G., J.L., D.T., J.U.M., M.M.L., S.M., C.L. Designed the experiments and analysed the data: S.J.G., C.L. Contributed reagents/materials/analysis tools: S.J.G., C.L. Statistical analysis: S.J.G., C.L. Wrote the paper: S.J.G., C.L. Critical revision of the manuscript: all authors.

## Data availability statement

Data are available from the corresponding author (C.L.) on reasonable request.
