# Supplementary material for: Proton pump inhibitor use and risk of hepatic encephalopathy: A multicentre study
Source: JHEP Rep. 2024 Apr 26;6(8):101104. doi: 10.1016/j.jhepr.2024.101104 (PMC11260370; doi:10.1016/j.jhepr.2024.101104)
Supplement: Multimedia component 1 [file mmc1.pdf]

Proton pump inhibitor use and risk of hepatic encephalopathy: A multicentre study

Simon Johannes Gairing, Chiara Mangini, Lisa Zarantonello, Elise Jonasson, Henrike Dobbermann, Philippe Sultanik, Peter Robert Galle, Joachim Labenz, Dominique Thabut, Jens Uwe Marquardt, Patricia P. Bloom, Mette Munk Lauridsen, Sara Montagnese, Christian Labenz

Table of contents

Supplementary figures 2

Supplementary tables 4

Supplementary figures

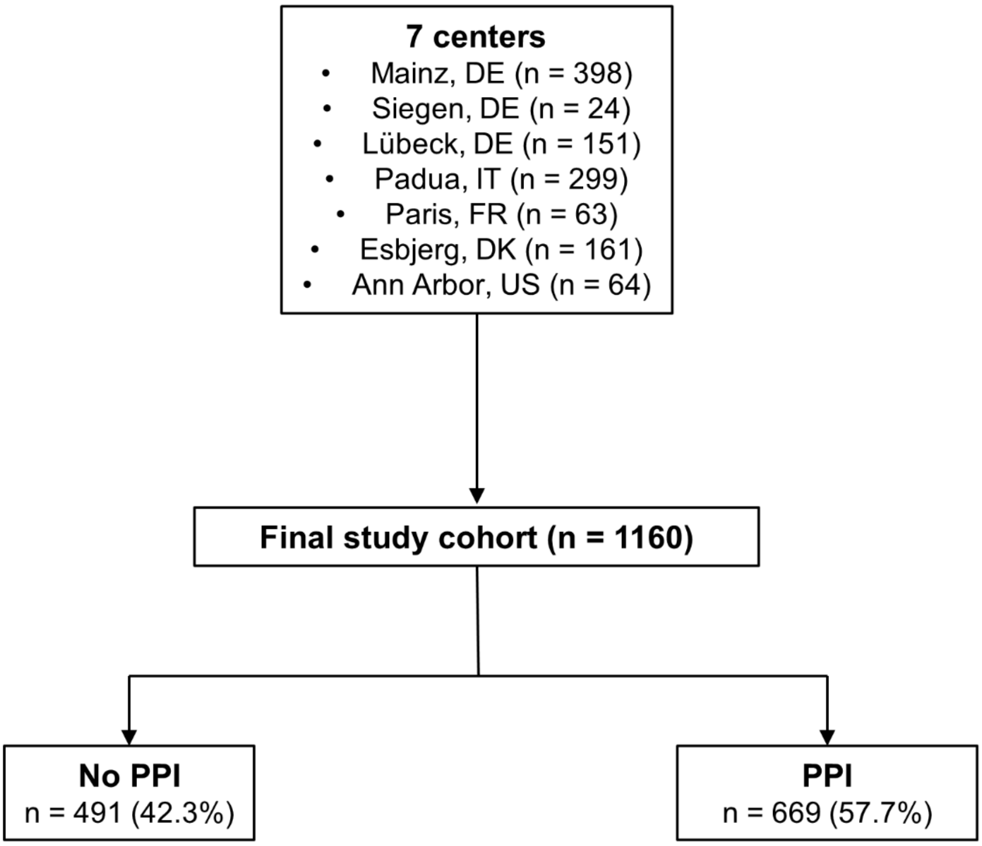

Fig. S1. Flowchart of enrolled patients. Abbreviations: DE, Germany; FR, France; IT, Italy; DK, Denmark; US, United States; PPI, proton pump inhibitors.

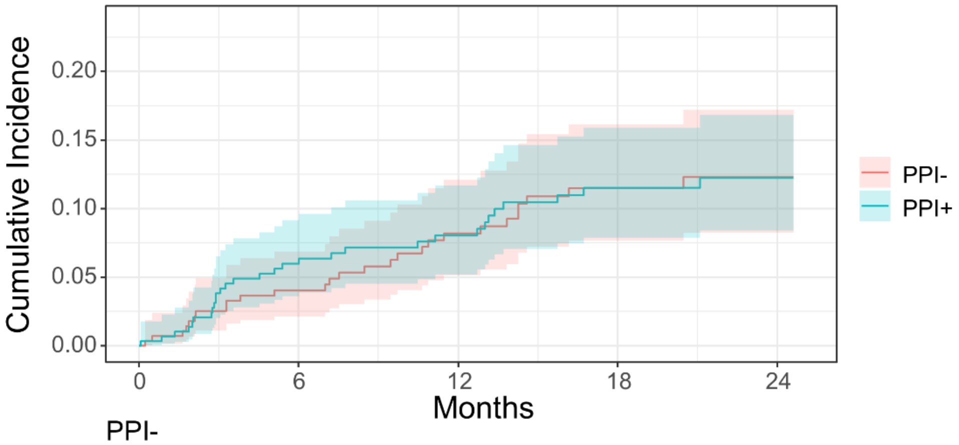

**PPI-**					
At Risk	280	234	171	116	71
Events	0	11	20	26	27
**PPI+**					
At Risk	296	242	183	141	81
Events	0	17	22	29	30

Fig. S2. Cumulative incidence of overt HE (OHE) stratified by PPI use in patients without a history of OHE and not taking lactulose and/or rifaximin. Abbr.: PPI, proton pump inhibitor.

Supplementary tables

Table S1. Demographics and clinical characteristics of patients without a history of overt HE.

	Total cohort (N=803)	PPI- (N=355)	PPI+ (N=448)
**Age (years)**			
Median [Min, Max]	60.0 [23.0, 87.0]	60.0 [23.0, 87.0]	60.0 [26.0, 86.0]
**Gender**			
male	502 (62.5%)	232 (65.4%)	270 (60.3%)
female	301 (37.5%)	123 (34.6%)	178 (39.7%)
**Etiology**			
Alcohol	375 (47.1%)	169 (47.7%)	206 (46.5%)
Viral	127 (15.9%)	61 (17.2%)	66 (14.9%)
Others/mixed	295 (37.0%)	124 (35.0%)	171 (38.6%)
Missing	6 (0.7%)	1 (0.3%)	5 (1.1%)
**MELD score**			
Median [Min, Max]	10.0 [6.00, 34.0]	10.0 [6.00, 29.6]	11.0 [6.00, 34.0]
Missing	34 (4.2%)	16 (4.5%)	18 (4.0%)
**Child Pugh**			
A	436 (56.3%)	217 (63.8%)	219 (50.5%)
B	259 (33.5%)	97 (28.5%)	162 (37.3%)
C	79 (10.2%)	26 (7.6%)	53 (12.2%)
Missing	29 (3.6%)	15 (4.2%)	14 (3.1%)
**ALBI grade**			
1	178 (29.9%)	98 (38.7%)	80 (23.4%)
2	310 (52.1%)	118 (46.6%)	192 (56.1%)
3	107 (18.0%)	37 (14.6%)	70 (20.5%)
Missing	208 (25.9%)	102 (28.7%)	106 (23.7%)
**MHE (PHES)**			
MHE-	582 (72.5%)	267 (75.2%)	315 (70.3%)
MHE+	221 (27.5%)	88 (24.8%)	133 (29.7%)
**History of ascites**			
no	396 (49.6%)	190 (53.8%)	206 (46.3%)
yes	402 (50.4%)	163 (46.2%)	239 (53.7%)
Missing	5 (0.6%)	2 (0.6%)	3 (0.7%)
**History of OHE**			
no	803 (100%)	355 (100%)	448 (100%)
yes	0 (0%)	0 (0%)	0 (0%)
**Sodium (mmol/L)**			
Median [Min, Max]	138 [116, 146]	139 [118, 145]	138 [116, 146]
Missing	115 (14.3%)	55 (15.5%)	60 (13.4%)
**Creatinine (mg/dL)**			
Median [Min, Max]	0.837 [0.370, 6.73]	0.820 [0.385, 2.44]	0.850 [0.370, 6.73]
Missing	43 (5.4%)	21 (5.9%)	22 (4.9%)
**Bilirubin (mg/dL)**			
Median [Min, Max]	1.10 [0.100, 33.3]	1.05 [0.175, 18.8]	1.17 [0.100, 33.3]
Missing	42 (5.2%)	19 (5.4%)	23 (5.1%)
**Albumin (g/L)**			
Median [Min, Max]	36.0 [15.0, 52.0]	37.6 [17.0, 52.0]	35.0 [15.0, 47.5]
Missing	128 (15.9%)	61 (17.2%)	67 (15.0%)
**INR**			
Median [Min, Max]	1.20 [0.800, 3.23]	1.20 [0.900, 3.20]	1.22 [0.800, 3.23]

	Total cohort (N=803)	PPI- (N=355)	PPI+ (N=448)
Missing	59 (7.3%)	28 (7.9%)	31 (6.9%)
**Lactulose**			
no	610 (76.0%)	293 (82.5%)	317 (70.8%)
yes	193 (24.0%)	62 (17.5%)	131 (29.2%)
**Rifaximin**			
no	713 (88.8%)	324 (91.3%)	389 (86.8%)
yes	90 (11.2%)	31 (8.7%)	59 (13.2%)
**Reason for PPI treatment**			
Reflux/GERD			61 (13.6%)
Ulcer disease			25 (5.6%)
Dyspepsia			21 (4.7%)
Ulcer prophylaxis			11 (2.5%)
Mixed/others			7 (1.6%)
**PPI indication**			
no			323 (72.1%)
yes			125 (27.9%)
**PPI type**			
Pantoprazole			283 (65.8%)
Esomeprazole			20 (4.7%)
Lansoprazole			52 (12.1%)
Omeprazole			65 (15.1%)
Rabeprazole			10 (2.3%)
No information on type			0 (0%)

Abbr.: MELD, model for end-stage liver disease; ALBI, albumin bilirubin ratio; MHE, minimal hepatic encephalopathy; OHE, overt hepatic encephalopathy; PPI, proton pump inhibitor.

Table S2. Demographics and clinical characteristics of patients stratified by center.

	Total cohort (N=1160)	Mainz (N=398)	Siegen (N=24)	Lübeck (N=151)	Padua (N=299)	Paris (N=63)	Esbjerg (N=161)	Ann Arbor (N=64)
**Age (years)**								
Median [Min, Max]	60.0 [23.0, 87.0]	60.0 [26.0, 87.0]	62.0 [34.0, 82.0]	61.0 [36.0, 87.0]	59.0 [23.0, 83.0]	61.0 [25.0, 82.0]	60.0 [27.0, 79.0]	63.0 [30.0, 81.0]
Missing	1 (0.1%)	0 (0%)	0 (0%)	0 (0%)	1 (0.3%)	0 (0%)	0 (0%)	0 (0%)
**Gender**								
male	758 (65.3%)	241 (60.6%)	14 (58.3%)	89 (58.9%)	229 (76.6%)	46 (73.0%)	104 (64.6%)	35 (54.7%)
female	402 (34.7%)	157 (39.4%)	10 (41.7%)	62 (41.1%)	70 (23.4%)	17 (27.0%)	57 (35.4%)	29 (45.3%)
**Etiology**								
Alcohol	553 (48.0%)	175 (44.3%)	14 (58.3%)	86 (57.0%)	101 (34.2%)	19 (30.2%)	133 (83.1%)	25 (39.1%)
Viral	174 (15.1%)	56 (14.2%)	1 (4.2%)	11 (7.3%)	87 (29.5%)	6 (9.5%)	5 (3.1%)	8 (12.5%)
Others/mixed	425 (36.9%)	164 (41.5%)	9 (37.5%)	54 (35.8%)	107 (36.3%)	38 (60.3%)	22 (13.8%)	31 (48.4%)
Missing	8 (0.7%)	3 (0.8%)	0 (0%)	0 (0%)	4 (1.3%)	0 (0%)	1 (0.6%)	0 (0%)
**MELD score**								
Median [Min, Max]	11.0 [6.00, 34.0]	10.2 [6.00, 27.0]	9.00 [6.00, 21.0]	10.3 [6.05, 29.6]	12.0 [6.00, 34.0]	12.0 [6.00, 33.0]	10.4 [6.43, 28.6]	10.0 [6.00, 20.0]
Missing	66 (5.7%)	0 (0%)	0 (0%)	6 (4.0%)	51 (17.1%)	0 (0%)	9 (5.6%)	0 (0%)
**Child Pugh**								
A	539 (49.4%)	225 (56.5%)	11 (45.8%)	77 (51.0%)	73 (31.3%)	23 (36.5%)	92 (57.9%)	38 (61.3%)
B	429 (39.4%)	134 (33.7%)	10 (41.7%)	56 (37.1%)	123 (52.8%)	36 (57.1%)	48 (30.2%)	22 (35.5%)
C	122 (11.2%)	39 (9.8%)	3 (12.5%)	18 (11.9%)	37 (15.9%)	4 (6.3%)	19 (11.9%)	2 (3.2%)
Missing	70 (6.0%)	0 (0%)	0 (0%)	0 (0%)	66 (22.1%)	0 (0%)	2 (1.2%)	2 (3.1%)
**ALBI grade**								
1	210 (24.8%)	91 (23.5%)	8 (40.0%)	27 (37.5%)	37 (18.2%)	11 (17.7%)	12 (30.8%)	24 (37.5%)
2	478 (56.4%)	206 (53.2%)	8 (40.0%)	40 (55.6%)	126 (62.1%)	42 (67.7%)	21 (53.8%)	35 (54.7%)
3	159 (18.8%)	90 (23.3%)	4 (20.0%)	5 (6.9%)	40 (19.7%)	9 (14.5%)	6 (15.4%)	5 (7.8%)
Missing	313 (27.0%)	11 (2.8%)	4 (16.7%)	79 (52.3%)	96 (32.1%)	1 (1.6%)	122 (75.8%)	0 (0%)
**MHE (PHES)**								
MHE-	775 (66.8%)	296 (74.4%)	8 (33.3%)	74 (49.0%)	225 (75.3%)	38 (60.3%)	103 (64.0%)	31 (48.4%)
MHE+	385 (33.2%)	102 (25.6%)	16 (66.7%)	77 (51.0%)	74 (24.7%)	25 (39.7%)	58 (36.0%)	33 (51.6%)
**History of ascites**								
no	506 (44.3%)	200 (50.3%)	8 (33.3%)	59 (39.1%)	112 (39.3%)	24 (38.1%)	75 (47.2%)	28 (45.2%)
yes	636 (55.7%)	198 (49.7%)	16 (66.7%)	92 (60.9%)	173 (60.7%)	39 (61.9%)	84 (52.8%)	34 (54.8%)
Missing	18 (1.6%)	0 (0%)	0 (0%)	0 (0%)	14 (4.7%)	0 (0%)	2 (1.2%)	2 (3.1%)

	Total cohort (N=1160)	Mainz (N=398)	Siegen (N=24)	Lübeck (N=151)	Padua (N=299)	Paris (N=63)	Esbjerg (N=161)	Ann Arbor (N=64)
**History of OHE**								
no	803 (70.6%)	351 (88.2%)	20 (83.3%)	129 (86.0%)	141 (48.1%)	21 (33.3%)	100 (68.0%)	41 (65.1%)
yes	335 (29.4%)	47 (11.8%)	4 (16.7%)	21 (14.0%)	152 (51.9%)	42 (66.7%)	47 (32.0%)	22 (34.9%)
Missing	22 (1.9%)	0 (0%)	0 (0%)	1 (0.7%)	6 (2.0%)	0 (0%)	14 (8.7%)	1 (1.6%)
**Sodium (mmol/L)**								
Median [Min, Max]	138 [116, 146]	139 [121, 146]	140 [128, 144]	138 [118, 146]	138 [116, 146]	139 [128, 146]	138 [127, 144]	139 [127, 144]
Missing	202 (17.4%)	2 (0.5%)	0 (0%)	0 (0%)	78 (26.1%)	0 (0%)	122 (75.8%)	0 (0%)
**Creatinine (mg/dL)**								
Median [Min, Max]	0.848 [0.320, 7.51]	0.830 [0.450, 2.23]	0.785 [0.370, 1.50]	0.916 [0.385, 6.73]	0.875 [0.320, 7.51]	0.781 [0.441, 3.05]	0.826 [0.452, 4.24]	0.885 [0.470, 2.21]
Missing	76 (6.6%)	7 (1.8%)	0 (0%)	0 (0%)	67 (22.4%)	0 (0%)	2 (1.2%)	0 (0%)
**Bilirubin (mg/dL)**								
Median [Min, Max]	1.24 [0.100, 33.3]	1.20 [0.290, 33.3]	1.05 [0.100, 14.7]	0.915 [0.170, 9.74]	1.64 [0.199, 32.4]	1.40 [0.175, 29.1]	1.22 [0.290, 8.82]	1.15 [0.300, 4.20]
Missing	78 (6.7%)	3 (0.8%)	0 (0%)	1 (0.7%)	72 (24.1%)	0 (0%)	2 (1.2%)	0 (0%)
**Albumin (g/L)**								
Median [Min, Max]	35.0 [14.0, 52.0]	35.0 [15.0, 46.0]	35.0 [24.0, 45.0]	36.5 [21.2, 51.2]	34.9 [20.0, 47.3]	34.0 [14.0, 45.0]	37.0 [21.0, 45.0]	39.0 [21.0, 52.0]
Missing	223 (19.2%)	11 (2.8%)	0 (0%)	0 (0%)	89 (29.8%)	1 (1.6%)	122 (75.8%)	0 (0%)
**INR**								
Median [Min, Max]	1.22 [0.800, 3.30]	1.20 [0.800, 3.30]	1.19 [0.900, 2.00]	1.20 [0.910, 2.76]	1.28 [0.990, 3.23]	1.34 [0.990, 2.23]	1.30 [0.900, 2.30]	1.10 [0.900, 3.20]
Missing	113 (9.7%)	7 (1.8%)	0 (0%)	6 (4.0%)	90 (30.1%)	0 (0%)	10 (6.2%)	0 (0%)
**Lactulose**								
no	723 (62.4%)	282 (70.9%)	12 (50.0%)	106 (70.2%)	127 (42.5%)	30 (47.6%)	132 (82.5%)	34 (53.1%)
yes	436 (37.6%)	116 (29.1%)	12 (50.0%)	45 (29.8%)	172 (57.5%)	33 (52.4%)	28 (17.5%)	30 (46.9%)
Missing	1 (0.1%)	0 (0%)	0 (0%)	0 (0%)	0 (0%)	0 (0%)	1 (0.6%)	0 (0%)
**Rifaximin**								
no	864 (74.5%)	365 (91.7%)	16 (66.7%)	127 (84.1%)	152 (50.8%)	30 (47.6%)	143 (89.4%)	31 (48.4%)
yes	295 (25.5%)	33 (8.3%)	8 (33.3%)	24 (15.9%)	147 (49.2%)	33 (52.4%)	17 (10.6%)	33 (51.6%)
Missing	1 (0.1%)	0 (0%)	0 (0%)	0 (0%)	0 (0%)	0 (0%)	1 (0.6%)	0 (0%)
**Reason for PPI treatment**								
Reflux/GERD	71 (10.6%)	28 (11.9%)	7 (36.8%)	11 (15.5%)	10 (5.1%)	2 (7.4%)	0 (0%)	13 (40.6%)
Ulcer disease	38 (5.7%)	6 (2.5%)	4 (21.1%)	5 (7.0%)	9 (4.6%)	0 (0%)	12 (13.8%)	2 (6.3%)
Dyspepsia	30 (4.5%)	0 (0%)	0 (0%)	0 (0%)	3 (1.5%)	0 (0%)	26 (29.9%)	1 (3.1%)
Ulcer prophylaxis	24 (3.6%)	2 (0.8%)	0 (0%)	3 (4.2%)	8 (4.1%)	7 (25.9%)	3 (3.4%)	1 (3.1%)

	Total cohort (N=1160)	Mainz (N=398)	Siegen (N=24)	Lübeck (N=151)	Padua (N=299)	Paris (N=63)	Esbjerg (N=161)	Ann Arbor (N=64)
Mixed/others	14 (2.1%)	3 (1.3%)	0 (0%)	2 (2.8%)	0 (0%)	4 (14.8%)	0 (0%)	5 (15.6%)
**PPI indication**								
no	492 (73.5%)	197 (83.5%)	8 (42.1%)	50 (70.4%)	167 (84.8%)	14 (51.9%)	46 (52.9%)	10 (31.3%)
yes	177 (26.5%)	39 (16.5%)	11 (57.9%)	21 (29.6%)	30 (15.2%)	13 (48.1%)	41 (47.1%)	22 (68.8%)
**PPI type**								
Pantoprazole	382 (59.7%)	214 (91.5%)	5 (26.3%)	62 (89.9%)	40 (23.3%)	5 (18.5%)	47 (54.0%)	9 (28.1%)
Esomeprazole	29 (4.5%)	1 (0.4%)	5 (26.3%)	1 (1.4%)	15 (8.7%)	4 (14.8%)	3 (3.4%)	0 (0%)
Lansoprazole	115 (18.0%)	5 (2.1%)	0 (0%)	0 (0%)	86 (50.0%)	15 (55.6%)	7 (8.0%)	2 (6.3%)
Omeprazole	100 (15.6%)	14 (6.0%)	0 (0%)	6 (8.7%)	27 (15.7%)	3 (11.1%)	30 (34.5%)	20 (62.5%)
Rabeprazole	13 (2.0%)	0 (0%)	9 (47.4%)	0 (0%)	4 (2.3%)	0 (0%)	0 (0%)	0 (0%)
No information on type	1 (0.2%)	0 (0%)	0 (0%)	0 (0%)	0 (0%)	0 (0%)	0 (0%)	1 (3.1%)

Abbr.: MELD, model for end-stage liver disease; ALBI, albumin bilirubin ratio; MHE, minimal hepatic encephalopathy; OHE, overt hepatic encephalopathy; PPI, proton pump inhibitor.

Table S3. Comparison of patients with vs without MHE.

	MHE-<br>(N=775)	MHE+<br>(N=385)	P-value
**Age (years)**			
Median [Min, Max]	60.0 [23.0, 87.0]	61.0 [27.0, 87.0]	0.05
Missing	1 (0.1%)	0 (0%)	
**Gender**			
male	486 (62.7%)	272 (70.6%)	0.009
female	289 (37.3%)	113 (29.4%)	
**Etiology**			
Alcohol	341 (44.4%)	212 (55.2%)	<0.001
Viral	135 (17.6%)	39 (10.2%)	
Others/mixed	292 (38.0%)	133 (34.6%)	
Missing	7 (0.9%)	1 (0.3%)	
**MELD score**			
Median [Min, Max]	10.0 [6.00, 27.0]	12.0 [6.00, 34.0]	<0.001
Missing	49 (6.3%)	17 (4.4%)	
**Child Pugh**			
A	411 (56.7%)	128 (35.1%)	<0.001
B	250 (34.5%)	179 (49.0%)	
C	64 (8.8%)	58 (15.9%)	
Missing	50 (6.5%)	20 (5.2%)	
**ALBI grade**			
1	166 (29.5%)	44 (15.5%)	<0.001
2	312 (55.4%)	166 (58.5%)	
3	85 (15.1%)	74 (26.1%)	
Missing	212 (27.4%)	101 (26.2%)	
**History of ascites**			
no	382 (50.0%)	124 (32.8%)	<0.001
yes	382 (50.0%)	254 (67.2%)	
Missing	11 (1.4%)	7 (1.8%)	
**History of OHE**			
no	582 (76.2%)	221 (59.1%)	<0.001
yes	182 (23.8%)	153 (40.9%)	
Missing	11 (1.4%)	11 (2.9%)	
**Sodium (mmol/L)**			
Median [Min, Max]	139 [118, 146]	138 [116, 146]	<0.001
Missing	145 (18.7%)	57 (14.8%)	
**Creatinine (mg/dL)**			
Median [Min, Max]	0.826 [0.370, 7.51]	0.905 [0.320, 6.73]	<0.001
Missing	61 (7.9%)	15 (3.9%)	
**Bilirubin (mg/dL)**			
Median [Min, Max]	1.15 [0.175, 33.3]	1.41 [0.100, 32.4]	0.001
Missing	64 (8.3%)	14 (3.6%)	
**Albumin (g/L)**			
Median [Min, Max]	36.0 [15.0, 52.0]	33.0 [14.0, 51.1]	<0.001
Missing	155 (20.0%)	68 (17.7%)	
**INR**			
Median [Min, Max]	1.20 [0.800, 3.30]	1.30 [0.900, 3.23]	<0.001
Missing	91 (11.7%)	22 (5.7%)	
**Lactulose**			

	MHE-<br>(N=775)	MHE+<br>(N=385)	P-value
no	537 (69.3%)	186 (48.4%)	<0.001
yes	238 (30.7%)	198 (51.6%)	
Missing	0 (0%)	1 (0.3%)	
**Rifaximin**			
no	622 (80.3%)	242 (63.0%)	<0.001
yes	153 (19.7%)	142 (37.0%)	
Missing	0 (0%)	1 (0.3%)	
**Reason for PPI treatment**			
Reflux/GERD	41 (9.6%)	30 (12.4%)	0.5
Ulcer disease	22 (5.2%)	16 (6.6%)	
Dyspepsia	18 (4.2%)	12 (5.0%)	
Ulcer prophylaxis	15 (3.5%)	9 (3.7%)	
Mixed/others	7 (1.6%)	7 (2.9%)	
**PPI indication**			
no	324 (75.9%)	168 (69.4%)	0.08
yes	103 (24.1%)	74 (30.6%)	
**PPI type**			
Pantoprazole	248 (60.2%)	134 (58.8%)	0.2
Esomeprazole	20 (4.9%)	9 (3.9%)	
Lansoprazole	79 (19.2%)	36 (15.8%)	
Omeprazole	60 (14.6%)	40 (17.5%)	
Rabeprazole	5 (1.2%)	8 (3.5%)	
No information on type	0 (0%)	1 (0.4%)	

Abbr.: MELD, model for end-stage liver disease; ALBI, albumin bilirubin ratio; MHE, minimal hepatic encephalopathy; OHE, overt hepatic encephalopathy; PPI, proton pump inhibitor.

Table S4. Multivariable Fine and Gray analysis for the development of OHE.

Variable	N	sHR	95% CI	p	N	sHR	95% CI	p	N	sHR	95% CI	p
	Total cohort				Child-Pugh A				Child-Pugh B			
**PPI use**												
no	381	—	—		214	—	—		133	—	—	
yes	527	1.08	0.77, 1.51	0.7	220	1.64	0.85, 3.16	0.14	237	1.03	0.65, 1.64	0.9
**MHE**												
no	599	—	—		331	—	—		211	—	—	
yes	309	1.28	0.90, 1.82	0.2	103	0.90	0.37, 2.18	0.8	159	1.72	1.08, 2.73	**0.022**
**MELD**	908	1.06	1.02, 1.09	**<0.001**	434	1.16	1.01, 1.34	**0.037**	370	1.05	0.99, 1.10	0.11
**Albumin (g/L)**	908	0.94	0.91, 0.96	**<0.001**	434	0.89	0.81, 0.98	**0.017**	370	0.97	0.93, 1.01	0.2
**History of OHE**												
no	661	—	—		362	—	—		229	—	—	
yes	247	1.76	1.19, 2.62	**0.005**	72	2.15	0.85, 5.42	0.11	141	1.50	0.91, 2.46	0.11
**Age (years)**	908	1.02	1.00, 1.03	**0.049**	434	1.00	0.97, 1.04	>0.9	370	1.02	1.00, 1.05	**0.034**
**Rifaximin**												
no	676	—	—		360	—	—		242	—	—	
yes	232	0.87	0.59, 1.29	0.5	74	1.68	0.70, 4.07	0.3	128	0.75	0.46, 1.23	0.3
**Lactulose**												
no	557	—	—		315	—	—		193	—	—	
yes	351	1.54	1.04, 2.29	**0.031**	119	1.98	0.88, 4.47	0.10	177	2.00	1.17, 3.42	**0.011**

Patients with missing data were excluded from analysis (complete case analysis). P-values in bold show significant values. Abbr.: sHR, subdistribution hazard ratio; CI, confidence interval; PPI, proton pump inhibitor; MHE, minimal hepatic encephalopathy; MELD, model for end-stage liver disease; OHE, overt hepatic encephalopathy.

Table S5. Univariable competing risk regression analysis for the development of OHE.

Variable	N	sHR	95% CI	p
**Age (years)**	1,159	1.00	0.99, 1.02	0.5
**Gender**				
male	758	—	—	
female	402	0.82	0.62, 1.09	0.2
**Etiology**				
Alcohol-associated	553	—	—	
Viral hepatitis	174	0.95	0.64, 1.41	0.8
Others / mixed	425	1.13	0.85, 1.49	0.4
**MHE (PHES)**				
no	775	—	—	
yes	385	1.74	1.34, 2.26	**<0.001**
**History of ascites**				
no	506	—	—	
yes	636	1.52	1.16, 1.98	**0.002**
**History of OHE**				
no	803	—	—	
yes	335	2.26	1.75, 2.93	**<0.001**
**MELD**	1,094	1.10	1.07, 1.12	**<0.001**
**Sodium (mmol/L)**	958	0.94	0.91, 0.98	**0.004**
**Creatinine (mg/dL)**	1,084	1.41	1.17, 1.69	**<0.001**
**Bilirubin (mg/dL)**	1,082	1.05	1.02, 1.08	**<0.001**
**Albumin (g/L)**	937	0.91	0.89, 0.93	**<0.001**
**INR**	1,047	2.28	1.64, 3.18	**<0.001**
**Lactulose**				
no	723	—	—	
yes	436	2.17	1.68, 2.81	**<0.001**
**Rifaximin**				
no	864	—	—	
yes	295	1.80	1.37, 2.37	**<0.001**
**PPI**				
no	491	—	—	
yes	669	1.23	0.94, 1.60	0.13
**PPI indication**				
no	492	—	—	
yes	177	0.92	0.63, 1.35	0.7

P-values in bold show significant values. Abbr.: sHR, subdistribution hazard ratio; CI, confidence interval; PPI, proton pump inhibitor; MHE, minimal hepatic encephalopathy; MELD, model for end-stage liver disease; OHE, overt hepatic encephalopathy.

Table S6. Multivariable competing risk regression analysis for the development of OHE.

Variable	N	sHR	95% CI	p
**PPI**				
no	374	—	—	
yes	517	1.16	0.82, 1.64	0.4
**MHE (PHES)**				
no	585	—	—	
yes	306	1.36	0.95, 1.95	0.091
**History of ascites**				
no	384	—	—	
yes	507	1.14	0.79, 1.64	0.5
**History of OHE**				
no	651	—	—	
yes	240	1.73	1.16, 2.59	**0.007**
**Sodium (mmol/L)**	891	1.02	0.96, 1.07	0.5
**MELD**	891	1.05	1.02, 1.09	**0.004**
**Albumin (g/L)**	891	0.94	0.91, 0.96	**<0.001**
**Lactulose**				
no	546	—	—	
yes	345	1.61	1.07, 2.43	**0.022**
**Rifaximin**				
no	666	—	—	
yes	225	0.84	0.55, 1.27	0.4

P-values in bold show significant values. Abbr.: sHR, subdistribution hazard ratio; CI, confidence interval; PPI, proton pump inhibitor; MHE, minimal hepatic encephalopathy; MELD, model for end-stage liver disease; OHE, overt hepatic encephalopathy.

Table S7. Multivariable Fine and Gray regression analysis for OHE development in patients without a history of OHE.

Variable	N	sHR	95% CI	p
**PPI use**				
no	288	—	—	
yes	373	1.24	0.75, 2.05	0.4
**MHE**				
no	467	—	—	
yes	194	1.87	1.18, 2.96	**0.008**
**MELD**	661	1.07	1.03, 1.12	**0.002**
**Albumin (g/L)**	661	0.92	0.89, 0.96	**<0.001**
**Age (years)**	661	1.02	1.00, 1.05	**0.041**
**Rifaximin**				
no	587	—	—	
yes	74	0.70	0.36, 1.39	0.3
**Lactulose**				
no	495	—	—	
yes	166	1.84	1.16, 2.91	**0.010**

Patients with missing data were excluded from analysis (complete case analysis). P-values in bold show significant values. Abbr.: sHR, subdistribution hazard ratio; CI, confidence interval; PPI, proton pump inhibitor; MHE, minimal hepatic encephalopathy; MELD, model for end-stage liver disease; OHE, overt hepatic encephalopathy.

Table S8. Multivariable Fine and Gray regression analysis for OHE development in patients without a history of OHE and not taking lactulose and/or rifaximin.

Variable	N	sHR	95% CI	p
**PPI use**				
no	225	—	—	
yes	243	0.91	0.47, 1.75	0.8
**MHE**				
no	352	—	—	
yes	116	1.75	0.88, 3.49	0.11
**MELD**	468	1.14	1.08, 1.21	**<0.001**
**Albumin (g/L)**	468	0.93	0.88, 0.98	**0.004**
**Age (years)**	468	1.03	1.00, 1.06	0.091

Patients with missing data were excluded from analysis (complete case analysis). P-values in bold show significant values. Abbr.: sHR, subdistribution hazard ratio; CI, confidence interval; PPI, proton pump inhibitor; MHE, minimal hepatic encephalopathy; MELD, model for end-stage liver disease; OHE, overt hepatic encephalopathy.
